# Emerging Functions of the Repeat Genome in Nuclear Structure: A View from the Human Karyotype

**DOI:** 10.1146/annurev-genom-111522-014017

**Published:** 2025-05-29

**Authors:** Lisa L. Hall, Kelly P. Smith, Jeanne B. Lawrence

**Affiliations:** Department of Neurology, University of Massachusetts Chan Medical School, Worcester, Massachusetts, USA

**Keywords:** human repeats, satellites, transposable elements, noncoding RNA, chromosome and nuclear structure, heterochromatin, euchromatin

## Abstract

Collectively, various tandem and interspersed repetitive sequences make up approximately half the human genome, yet we have only begun to understand the potential functions of “junk” DNA. Here, we provide a brief overview of various types of repeats, but a full treatment of the repeat genome (repeatome) is beyond the scope of any review. Hence, we focus primarily on less established functions of a few major repeat classes, including pericentromeric satellites and abundant degenerate interspersed repeats, short interspersed nuclear elements (Alu), and long interspersed nuclear elements (L1). A theme developed throughout is how sequence organization in the human karyotype provides insights into potential functions within nuclear structure. For example, millions of small tandem major satellite repeats can form bodies that sequester nuclear factors, or the segmental organization of interspersed repeats may underpin the nuclear compartmentalization of heterochromatin and euchromatin. Decoding the vast repeatome is an exciting frontier being enabled by recent technological advancements. However, identifying the extent of meaningful information in repeats will likely require concepts that go well beyond impacts for individual genes, to new ways to identify and interpret broad patterns of genome-wide organization and nucleus-wide regulation.

## 1. INTRODUCTION

The Human Genome Project initially focused on sequencing the ~20,000 protein-coding genes, and there was debate as to whether the rest of the genome, riddled with repetitive sequences, was worthy of the cost and time to interrogate it. However, we now know that polymorphisms of interest are often in repeat-rich noncoding regions. Repetitive sequences have been notoriously difficult to investigate and map and thus have often been screened out of analysis using RepeatMasker (https://www.repeatmasker.org) or similar programs. Recent advances in long-read sequencing technology make it possible to read through and precisely map sequences in large repetitive regions, allowing comprehensive chromosome sequencing in the Telomere-to-Telomere (T2T) project (83, 127). This has produced the first full, contiguous sequencing of the repeatome, including large satellite regions, revealing greater structural complexity than previously anticipated. Multiple studies have begun to extend this technology to create pangenome reference sequences that capture polymorphic differences in various repeats in populations (reviewed in 155). As we obtain a more complete description of the human genome’s repeat content and its variations, a major challenge will be to assess the potential impacts of variation in different types of repeats, and to understand the extent to which various types of repetitive “junk” play any functional roles.

To undertake a review on the emerging functions of the various abundant repeats in the human genome is a timely but dauntingly large task. A full accounting of human repeats is beyond the scope of any review, and we apologize that we cannot fully represent the huge literature of related work. Here, we present a conceptual overview of emerging functions, focusing largely on a few of the most abundant repeats with less established functions. Gene regulation has been most studied in terms of local sequence effects on individual genes, and repeat elements often function at that level. However, we will convey our perspective that highly abundant repeats may also function more collectively, to influence regional genome regulation in nuclei. This may best be understood through the lens of human genome organization on chromosomes and how it relates to compartmentalized genome regulation within complex nuclear structure.

### 1.1. Half the Human Genome Is Composed of Different

#### Types of Repeat Sequences

**Figure 1:**
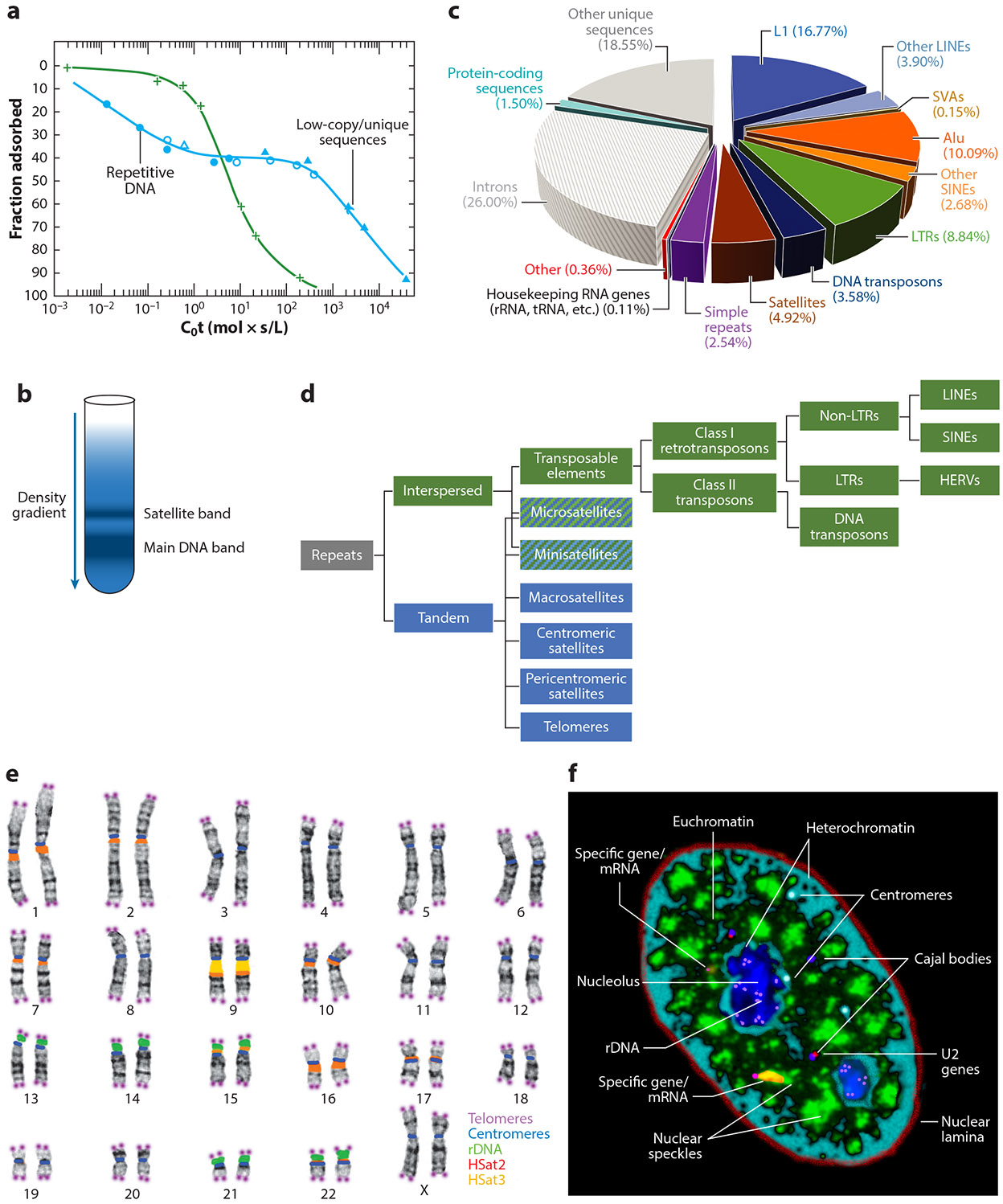
(*a*) Britten & Kohne’s (20) graph of the renaturation kinetics of calf thymus DNA (*blue circles* and *triangles*), showing ~40% rapidly reannealing repetitive sequences and ~50–60% more unique sequences. *Escherichia coli* DNA (*green plus signs*) lacks the highly repetitive fraction. Panel adapted with permission from Reference 20. (*b*) Density gradient centrifugation separating a smaller satellite band from the main band of genomic DNA. (*c*) Pie chart showing the relative abundances of repeat types in the human genome. Data are from Reference 83. (*d*) Categories of repeat types. Note that not all repeat types in each category are shown here. (*e*) Karyotype showing alternating light and dark bands on a G-banded mitotic chromosome spread, with the locations of several repeat sequence types indicated. Panel adapted with permission from Reference 145. (*f*) Illustration depicting the organization of the interphase nucleus. The genome has a compartmentalized architecture within the nucleus and is organized further relative to non-membrane-bound substructures rich in RNA metabolic factors, as indicated. Panel adapted with permission from Reference 145. Abbreviations: HERV, human endogenous retrovirus; HSat, human satellite; L1, long interspersed nuclear element 1; LINE, long interspersed nuclear element; LTR, long terminal repeat; SINE, short interspersed nuclear element; SVA, SINE-VNTR-Alu; VNTR, variable number tandem repeat.

More than 50 years ago, DNA reannealing experiments discovered that large portions of the genomes of higher organisms are composed of highly or moderately repeated sequences (20). The classical C_0_t curves (Figure 1a) show that abundant repeats reanneal at the lowest concentration and time (C_0_t-1), while unique or low-copy sequences reanneal more slowly. Subsequent studies indicated that much of this repetitive DNA was interspersed throughout the genomes of many organisms, although the proportion of the genome occupied by repeats can vary widely (41, 69). More than half a century ago, Britten & Davidson (19) theorized that these sequences may function to regulate the genome. A few years earlier, cesium chloride density gradient centrifugation studies of DNA from several species had resolved a primary band of DNA and a smaller satellite band (97) (Figure 1b). This DNA satellite was subsequently shown to comprise large tandem arrays of short repeated sequences (165) that are AT rich (37) and localized mostly at or near chromosome centromeres (89).

Notably, half the human genome is composed of various repetitive sequences (Figure 1c), which can be categorized as one of two major types: tandem repeats, which localize to specific chromosomal sites, or interspersed repeats, which distribute widely through chromosomes, mostly as single repeat units (Figure 1d). As discussed in Section 3, most interspersed repeats were derived from mobile transposable elements (TEs) that invaded the human genome, but why degenerate forms of TEs remain so abundant is an unsolved question in genome biology.

While the potential biological significance for the bulk of repetitive sequences is not known, numerous studies have shown that a specific repeat sequence, typically near or in a protein-coding gene, can impact the function of that gene through numerous different mechanisms, which can be mediated by DNA or RNA. Changes in the location or copy number of a repeat can have deleterious effects and contribute to disease or can be co-opted during evolution to contribute to normal gene function. While there are now many examples of a repeat sequence being co-opted to impact local gene function, they do not necessarily indicate whether the bulk of highly abundant degenerate repeats contribute to genome function more broadly or if they are just an evolutionary vestige. We discuss here less established concepts for how certain repeat types, present in enormous numbers (hundreds of thousands to a million), may contribute to the broader regulation of the genome within nuclear structure.

Identifying and understanding novel mechanisms may require different conceptual approaches that go beyond the better-known molecular mechanisms that regulate individual genes. Adding to the challenge, certain repeat types may function only transiently at particular stages of early development, in response to stress, or in specific disease states, examples of which are mentioned throughout this review. We consider these functions from the perspective of repeat genome organization in the human karyotype, which we suggest can provide insight into potentially broader collective roles of abundant repeats in genome regulation.

### 1.2. Location, Location, Location: Connecting Chromosomal Organization and Nuclear Genome Function

As the title suggests, this review discusses repeat sequences with “a view from the human karyotype,” with the underlying premise that the higher-order chromosomal distribution of repetitive sequences (and genes) has been shaped through evolution to facilitate function. While the locations of telomeric or centromeric repeats clearly reflect their specific roles in chromosome structure (discussed below), the linear sequence organization on chromosomes can also relate to their function within the nucleus. The most straightforward demonstration of this is that tandem copies of rDNA genes (and associated satellite repeats) occupy the small short arms of all five acrocentric human chromosomes (Figure 1e). In our view, this singular organization evolved to facilitate the formation of the nucleolus, a factory that promotes highly efficient rRNA transcription, processing, and assembly of the numerous components required to produce abundant ribosomes. Several other examples illustrate this principle on a smaller scale; for example, the clustering of tandemly repeated U2 small nuclear RNA genes facilitates their association with the nuclear Cajal body involved in the assembly of small nuclear ribonucleoproteins. Hence, the organization of DNA sequences, and often the RNAs they produce, can nucleate efficient nuclear hubs for complex functions, which we previously referred to as the karyotype-to-hub hypothesis (145). This principle is also relevant here as we consider the complexity and puzzling abundance of nongenic repetitive sequences throughout the genome.

The human genome is full of larger cytogenetic patterns, revealing sequence organization so pronounced that it is evident from simple staining and light microscopy. In addition to numerous and often huge pericentric satellites, staining shows a pattern of 400–600 alternating light and dark Giemsa-stained bands (Figure 1e), which correspond largely to regions with differences in gene density, short interspersed nuclear elements (SINEs) versus long interspersed nuclear elements (LINEs), and GC versus AT content. What might be the functional significance of these cytological-scale differences in linear genome organization? We suggest that a full understanding will require a perspective on the complex substructure of the interphase nucleus, including the compartmentalization of euchromatin and heterochromatin into large distinct nuclear regions, and in cell type–specific patterns (Figure 1f). The large euchromatin compartment, which is typically more internal in the nucleus, is punctuated by ~10–20 discrete nuclear speckles (also known as SC35 domains) that are concentrated with a host of RNA metabolic factors. This will become important in Section 4 when we consider the large segmental organization of the genome, as reflected in chromosome bands, with differences in the density and types of genes as well as repetitive sequences.

**Figure 2:**
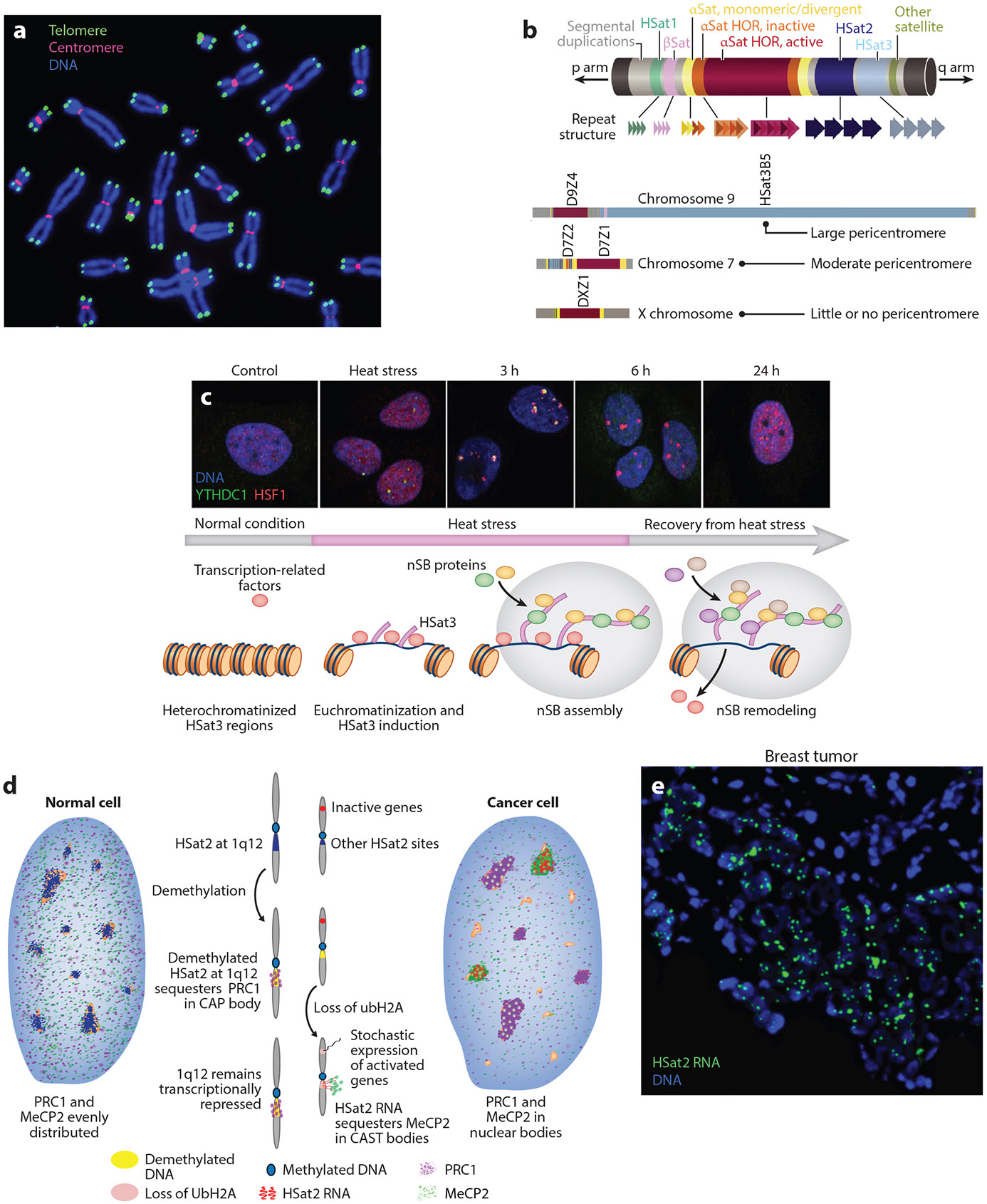
(*a*) Digital image of human telomeres and centromeres on metaphase chromosomes detected by FISH using labeled oligonucleotide probes for telomeric (*green*) and centromeric (*red*) sequences and DAPI DNA dye (*blue*). Image provided by the laboratory of Dr. Jerry W. Shay. (*b*) Schematic of a generalized human peri/centromeric region. The amount of pericentromeric sequence varies greatly among different chromosomes. Panel adapted with permission from Reference 3. (*c*) nSB formation. (*Top*) YTHDC1 (*green*) and HSF1 (*red*) fluorescent immunostaining and DAPI DNA staining (*blue*) in control cells, recruitment of HSF1 upon heat stress, sequestration of YTHDC1 at 3 and 6 h during recovery, and return to normal after 24 h. (*Bottom*) Diagram of HSat3 expression and nSB formation. HSat3 repeats, which are normally heterochromatin, become expressed upon heat stress. HSat3 RNA recruits specific proteins, assembling nSBs, which are subsequently remodeled through recruitment of other factors. Top subpanel adapted from Reference 156 (CC BY 4.0); bottom subpanel adapted from Reference 124 (CC BY 4.0). (*d*) Schematic of CAP and CAST body formation by HSat2. In many tumors, DNA demethylation triggers HSat2 DNA and RNA molecular sponges, causing further epigenetic dysfunction. Sequestration of PRC1 at the demethylated 1q12 megasatellite forms CAP bodies and reduces the repressive ubH2A modification at other HSat2 loci, which express RNA that sequesters MeCP2 in CAST bodies. Panel adapted with permission from Reference 72 (CC BY-NC-ND 4.0). (*e*) Image showing HSat2 RNA (*green*) forming CAST bodies in breast tumor cells (with DAPI-stained nuclear DNA shown in *blue*). Panel adapted with permission from Reference 72 (CC BY-NC-ND 4.0). Abbreviations: αSat, alpha satellite; βSat, beta satellite; CAP, cancer-associated Polycomb; CAST, cancer-associated satellite transcript; DAPI, 4′,6-diamidino-2-phenylindole; FISH, fluorescence in situ hybridization; HOR, higher-order repeat; HSat, human satellite; nSB, nuclear stress body; ubH2A, ubiquitinated histone H2A.

As alluded to above, the organization of telomere repeats (Figures 1e and 2a) clearly reflects their function, to cap each chromosome end and protect it from fusing with other chromosomes (see 28). However, telomere biology also illustrates that a given repeat sequence can have more than one function and that the study of repeats can reveal unanticipated and fundamentally important biology. The discovery that attrition of the telomere array is essentially a cellular aging clock fueled numerous important discoveries in developmental biology and disease, particularly cancer (reviewed in 6). Arrays of the telomere repeat TTAGGG are several kilobases in newborns and are protected by the shelterin complex (reviewed in 43), but telomeres shorten progressively with each somatic cell division; when they reach a critical length, a DNA damage response then triggers cell senescence. In pluripotent cells, telomere length is maintained by telomerase, an enzyme largely absent in differentiated cells, leading to telomere shortening and cell senescence (reviewed in 28, 55). This example affirms the compelling prospects to uncover important new biology by mining for meaningful information in the complex dark matter of human repetitive sequences.

## 2. EMERGING ROLES OF PERICENTRIC SATELLITES IN NUCLEAR STRUCTURE

Tandem repeats vary greatly in terms of size of the repeat unit and length of the array. Categorized largely by array size, satellites are the largest, followed by macro-, mini-, and microsatellites, with some overlap between category definitions (see the sidebar titled Categories of Human Tandem Repeats). We briefly discuss these smaller satellite types before focusing on the very large major satellite arrays, especially those without a known or established function.

### 2.1. Many Mini- and Microsatellite Repeats Can Impact the Functions of Specific Disease-Associated Genes in *Cis*

Diverse short tandem repeats (STRs) are present at many loci across our genomes, and changes in individual tandem repeats can cause dysfunction in specific disease-associated genes. Approximately 50 monogenic diseases have been linked to the expansion or contraction of STRs (mostly triplet repeats) in or near disease-causing genes, which can produce gain- or loss-of-function effects on normal genes. These are primarily neurological diseases such as Huntington disease (CAG), fragile X syndrome (CGG), myotonic dystrophy (CTG), Friedreich ataxia (GAA), spinocerebellar ataxia (CAG), and amyotrophic lateral sclerosis (reviewed in 47).

These repeats are also thought to play a variety of roles in normal gene function (reviewed in 7), including operating as part of gene products [e.g., in coding exons (151) or noncoding RNA (ncRNA) functional domains (21)], by influencing local chromatin structure and transcription [e.g., nucleosome spacing, CpG methylation, transcription factor (TF) binding sites, transcription start sites, and enhancers] and acting within untranslated regions or introns to modulate transcription, translation, and alternative splicing. These highly variable arrays provide a larger polymorphic range than the more binary single-nucleotide polymorphisms, and numerous studies implicate these repeats in many common human disorders, including neurological and neurodegenerative disorders, cardiovascular disease, diabetes, and cancer (reviewed in 47, 77, 117). Therefore, STRs could contribute to the missing heritability in multifactorial conditions and in normal phenotypic variation. And with recent advances in sequencing technology, their true variation is now being explored (e.g., 137).

We highlight just one of the first triplet repeat disorders discovered, which illustrates a theme developed further below for major satellites: the capacity of very abundant small repeats to bind and sequester regulatory factors. Myotonic dystrophy type 1 results from a large expansion of CTG triplet repeats in the 3′ untranslated region of the *DMPK* gene. This causes the *DMPK* mRNA containing the repeats to accumulate to high levels in the nucleus, forming ribonucleoprotein aggregates that sequester an important splicing regulator, MBNL (muscleblind-like). MBNL levels throughout the nucleoplasm drop sharply as a consequence (144, 167), which impairs alternative splicing of pre-mRNAs for many other genes (reviewed in 111). The concept that highly abundant repeats can bind and impact the distribution of specific nuclear factors will be important as we consider the function of the much larger tandem repeats, satellites.

### 2.2. Large Major Satellite Arrays of Small Repeats at Specific Loci: Not-So-Constitutive Heterochromatin

The much larger major satellites are very different in form and function than the smaller tandem repeats discussed above, as these huge arrays are located predominantly at one location: at or near the centromeres of chromosomes. The three major satellites we discuss here—alpha satellite (αSat), human satellite 2 (HSat2), and HSat3—are multi-megabase arrays comprising tens or hundreds of thousands of small repeats at a single locus. Historically thought to be constitutively silent heterochromatin, large satellites are now recognized to be transiently expressed in different contexts during early embryonic development, cell cycle stages, specific diseases (such as cancer), or changes in cell state such as stress (discussed in Section 2.4.1). The transient and complex nature of satellite expression patterns will make it more challenging to fully investigate their biological functions, but results to date make clear that they can serve important functions at the DNA and/or RNA level.

Why did the genome evolve to accumulate many thousands or millions of tandem copies of a small sequence in singular locations? All of these large satellites are in or adjacent to the centromere, and we begin by discussing the more well-established role for αSat in centromere function. The functions of pericentric satellites (HSat2 and HSat3) are less clear: While they may play a structural role related to centromere function, we highlight emerging evidence that they can also play a role in global genome regulation in nuclei.

### 2.3. Centromeric αSat Repeats Localize Proteins to Form the Kinetochore of Segregating Chromosomes

Centromeres (Figure 2a,b) are composed of a 171-bp αSat repeat unit in a single 2–5-Mb array on each chromosome, producing a structure essential for kinetochore assembly and function during mitosis and meiosis. Centromeric satellite DNA has both active and inactive histone marks and is transcribed at low levels in human cells. The transcripts form DNA:RNA hybrids that stabilize an RNA:protein structure at the locus that facilitates recruitment and stabilization of most centromeric proteins [centromere proteins (CENPs), passenger complex, etc.] required for centromere function (reviewed in 172). Preventing centromere transcription leads to gradual loss of these centromeric proteins and genomic instability (reviewed in 36).

The accumulation of CENP-A (a histone H3 variant) in nucleosomes uniquely distinguishes the centromere proper from the rest of the genome, and this chromatin serves as the platform for kinetochore assembly, an enormous complex that binds spindle microtubules during cell division (reviewed in 91). The accumulation of ~100 different proteins on αSat centromeric DNA illustrates how high-copy repeats organized into arrays serve to concentrate protein components to build a structure that functions at that site.

αSat array size and sequence polymorphisms have been associated with defective centromere architecture and aneuploidies, and polymorphic satellite array size can vary between homologs (reviewed in 121, 152). This suggests that these polymorphic differences may play important roles in human health, but until recently, human centromeres were almost entirely absent from the genome build, hindering their study. The T2T gapless assembly published in 2022 for the first time includes all human centromeric sequences, and the inclusion of diverse populations has revealed more variability than expected in αSat sequences, especially among people of recent African origin (3).

### 2.4. Diverse Functions for the Huge Pericentric Satellites: HSat2 and HSat3

Pericentric satellites adjacent to the centromere (Figure 2b) were nearly absent from the reference human genome until recently (2), making them significantly understudied. The two most abundant are HSat2 and HSat3, which total 28.7 and 47.6 Mb, respectively, and are found on numerous, but not all, human chromosomes. HSat3 is derived from a pentameric repeat, (CATTC)_*n*_, and the HSat2 repeat is an ~26-bp degenerate sequence derived from the HSat3 pentamer. Together, they constitute the largest contiguous satellite arrays in the human genome, including an ~28-Mb HSat3 array on chromosome 9 and the two largest HSat2 arrays, on chromosomes 1 and 16, which are approximately half that size (~14 Mb) (2) (Figure 1e).

Pericentric satellites are generally silent in most normal cells, with the exception of testis and brain, and their heterochromatic nature may help stabilize the centromere (reviewed in 60). However, not all human chromosomes have pericentric satellites (Figure 2b), which indicates that they are not necessarily required for normal centromere function. Nevertheless, aberrations in their heterochromatic state or expression have been associated with mitotic defects in spindle attachments and sister chromatid cohesion, as well as increased DNA damage in S-phase due to blocked replication over pericentric DNA:RNA hybrids (reviewed in 146).

Interestingly, however, pericentric satellites harbor promoter elements that can regulate transcription by RNA polymerase II (RNAPII) or RNAPIII, and pericentric satellite expression is common during embryogenesis. In fact, many different human satellite families are expressed in complex patterns during early embryogenesis that appear to be highly regulated (reviewed in 120, 146). This implies directed regulation of individual satellite arrays during specific windows of embryonic development, for currently unknown reasons. One possibility is that pericentric satellite expression early in embryogenesis plays a role in nucleating the formation of initial heterochromatic compartments with this unique chromatin (reviewed in 133). However, this has yet to be fully explored, as these regions are not currently included in the genome maps for most species and have only recently been added to the human genome build.

Another possible function is that both HSat2 and HSat3 can impart global gene regulation through their capacity to act as a cytological-scale molecular sponge. The collective evidence summarized below suggests that these exceptionally high-copy pericentric arrays, containing repeat units with protein-binding potential, have extraordinary capacity to amass and cytologically sequester regulatory factors at both the DNA and RNA levels, thereby modifying their accessibility on a genome-wide scale. For example, a 14-Mb array of a 26-nt repeat unit will contain ~500,000 copies, while a 5-nt sequence could be repeated millions of times in a single multi-megabase location. Hence, there is a remarkable potential to congregate or sequester factors at one location within nuclear structure. Interestingly, centric satellite repeats function similarly in various species, yet the satellite sequence itself is not conserved (e.g., between human and mouse). This illustrates that the function of repeats is often less stringently tied to primary sequence than it is for protein-coding genes.

#### 2.4.1. HSat3 DNA and RNA: the nuclear stress sponge.

The earliest and most developed evidence of a human satellite functioning as a sponge is for the very large HSat3 array on chromosome 9 (9q12) and more recently recognized on the Y chromosome. These arrays act at both the DNA and RNA levels to regulate cell homoeostasis during numerous types of cell stress (e.g., heat shock, osmotic or oxidative stress, and UV radiation). Cell stress triggers a series of steps in which different factors are sequentially bound and released from HSat3 DNA or RNA during the stress response, including a prolonged recovery process (reviewed in 61, 124) (Figure 2c). At stress onset, *HSF1* (heat shock transcription factor 1) is expressed, and the HSF1 protein localizes to the normally silent HSat3 loci on chromosome 9 and the Y chromosome, along with several other TFs and chromatin-remodeling factors. HSF1 activates HSat3 transcription via RNAPII, and the HSat3 ncRNA transcripts accumulate at the locus, forming ribonucleoprotein bodies called nuclear stress bodies. Nuclear stress bodies sequester many different RNA metabolic factors, leading to global suppression of transcription and translation, until the stress is resolved. During stress recovery, these components are released to the nucleoplasm to reactivate the genome in a highly regulated manner. Nuclear stress bodies remain during the prolonged stress recovery period and sequester other factors to help reverse the process, suggesting that the same bodies can dynamically change their properties and function. In the final stage of stress recovery, the remaining HSat3 transcripts recruit repressive factors to re-silence the HSat3 loci, making the sponge inert once again.

The ability of repetitive RNA to nucleate phased domains confers a second means to affect genome regulation broadly: by concentrating specific factors together in a reaction crucible that accelerates biochemical reactions. HSat3 nuclear stress bodies also behave this way during cell stress (reviewed in 124), illustrating the versatility of satellite RNA bodies in broadly regulating the genome.

#### 2.4.2. HSat2 DNA and RNA: the nuclear disease sponge.

The large HSat2 arrays on chromosomes 1 and 16 are two of the most prominent but poorly studied features of the human genome. Recent studies have uncovered unanticipated biology of HSat2 satellites that points to effects mediated by DNA demethylation and RNA expression, but with complex differences between HSat2 loci on different chromosomes. Translocations and duplications of the large HSat2 array at 1q12 are among the most frequent aberrations in cancers (reviewed in 70), and global DNA demethylation is also common in human cancers [and in ICF (immunodeficiency, centromeric region instability, and facial anomalies) syndrome (160)], with HSat2 at 1q12 being especially sensitive to demethylation (53).

This global demethylation appears to trigger HSat2 at 1q12 to act as a molecular sponge (72), which further alters the epigenetic state of the cell. DNA demethylation causes PRC1 (Polycomb repressive complex 1), which normally maintains the repressive ubiquitinated histone H2A (UbH2A) mark at target gene loci across the genome, to accumulate over the 1q12 locus, forming large cancer-associated Polycomb (CAP) bodies (72) (Figure 2e). Although several human chromosomes have smaller pericentromeric HSat2 arrays, this is a locus-specific (1q12) and protein-specific (PRC1 but not PRC2) response to DNA demethylation in human cells, while in mouse, both PRC1 and PRC2 (which trimethylates H3K27) are sequestered to all major satellites in pericentromeres upon demethylation (33). This difference likely reflects the greater chromosome-specific sequence diversity in human satellites compared with mouse pericentric satellites (84, 162) and suggests that humans evolved locus-specific satellite function.

The functional sequestration of epigenetic factors to large pericentromeric arrays can have downstream consequences for global transcriptional regulation, which results in aberrant expression across the genome (33, 72). Importantly, this includes small HSat2 arrays on other chromosomes, which become derepressed and aberrantly expressed (72) (Figure 2d). In fact, numerous studies have shown that normally silent HSat2 repeats are commonly overexpressed in cancers, more frequently than any other satellite (13, 72, 96), as well as during viral infection, senescence, and DNA damage and in diseases like facioscapulohumeral muscular dystrophy (e.g., 125, 141).

Aberrant expression from HSat2 loci then compounds the epigenetic dysregulation in these cells (Figure 2d) by sequestering additional regulatory factors into large ribonucleoprotein bodies. These HSat2 RNA bodies are prominent hallmarks of many tumors (Figure 2e), detected in approximately half of 34 diverse tumors examined, and sequester MeCP2 (methyl-CpG binding protein 2) (72, 102). These were initially termed cancer-associated satellite transcript (CAST) bodies; however, HSat2 RNA bodies are also seen in other disease contexts, where we call them satellite transcript (SATT) bodies, and sequester different regulatory factors, including CTCF (CCCTC-binding factor) (122), EIF4A3 (eukaryotic translation initiation factor 4A3), and ADAR1 (adenosine deaminase RNA 1) (141), leading to further dysregulation of cell homeostasis and gene expression. Thus, HSat2, like HSat3, can form both DNA and RNA molecular sponges that impact genome-wide access to important regulatory factors and broadly affect gene expression.

#### 2.4.3. HSat2 expression in development and disease and potential regulatory effects on gene pathways.

Aberrant expression of satellites in disease may not only be a consequence of misregulation but also directly contribute to effects on specific downstream pathways. There is some evidence to suggest that cancer cells or viruses co-opt HSat2 expression to impact pathways that confer a growth advantage. For example, tumors appear to select for expression of specific satellites (72, 148), and many human viruses use TFs to specifically activate HSat2 loci (126). The presence of these satellite RNAs (particularly HSat2) is associated with gene expression changes that confer reduced immune response, changes in cellular motility, or changes in protein stability and localization (e.g., 126, 134, 148). And some work has also shown a direct link between the presence of the HSat2 ncRNA and aberrant expression from specific pathways (loss of the RNA prevented the effect) (126). This suggests that HSat2 RNA itself altered the regulation of specific gene pathways via an unknown mechanism, which may well be through sequestration of their regulatory factors. However, most studies do not look for expression from the repeatome or sequestration to RNA bodies, so it is unclear whether SATT bodies are responsible for the gene expression changes observed.

Most human satellite families are enriched in a wide variety of satellite-specific TF binding sites, including those that regulate conserved signaling pathways (62, 162). Since expression of different satellite families appears to be highly regulated throughout embryogenesis, and global hypomethylation of satellites is also a normal hallmark of gametes, preimplantation embryos, and extraembryonic tissues (reviewed in 163), it is tempting to speculate that satellites may act as DNA or RNA molecular sponges at important transition points during normal development. This may dynamically regulate global genomic access to specific regulatory factors during developmental transitions.

The likelihood that satellites can act as DNA or RNA sponges during embryogenesis is supported by findings that mouse pericentromeres (which form chromocenters) can functionally sequester specific TFs during mouse embryogenesis (110). Additionally, the DUX4 (double homeobox 4) TF is expressed during specific windows of human embryogenesis [and by many human cancers and viruses (reviewed in 123)] and is required for proper maturation of pre- and postimplantation embryos (141, 161). DUX4 induces expression of HSat2 (and other repeats) during these same developmental windows, with HSat2 SATT body formation and sequestration of key regulators.

The fascinating story of DUX4 began with the discovery of its abnormal activation in facioscapulohumeral muscular dystrophy (50) and illustrates the importance of including the repeatome in both transcriptomic and molecular cytology studies (reviewed in 123). The macrosatellite D4Z4, on the subtelomere of chromosome 4q, is heterochromatic and silent in most adult cell types. However, reducing the copy number of the 3.3-kb repeat in the D4Z4 array to <10 triggers loss of heterochromatic repression and aberrant DUX4 expression, which is considered the primary cause of muscle degeneration (71). DUX4, which normally regulates HSat2 expression during development, induces aberrant HSat2 RNA in facioscapulohumeral muscular dystrophy muscle. HSat2 SATT bodies in cell nuclei sequester important regulatory factors that affect RNA stability, splicing, and translation. Hence, future studies will need to consider what downstream consequences are directly due to the DUX4 TF or, alternatively, might be due to effects of sequestration of regulatory factors by HSat2.

This example illustrates how a macrosatellite can act locally to regulate the epigenetic state of a locus (4q35) that encodes an important regulatory gene (*DUX4*), which in turn normally regulates embryonic expression of a major satellite (HSat2), potentially affecting nuclear regulatory factors more globally.

## 3. ABUNDANT INTERSPERSED REPEATS DERIVED FROM MOBILE TRANSPOSABLE ELEMENTS

The largest portion of the repeatome consists of interspersed repeats derived from TEs that long ago invaded the human genome and influenced its evolution. TEs are mobile DNA sequences capable of transposition and integration into the genome and can be divided into two broad categories based on their mechanism of transposition. Class I TEs are retrotransposons, which mobilize by transcription into an RNA intermediate and then reverse transcription into a new genomic location, while class II TEs are DNA transposons that move as DNA via a variety of cut-and-paste mechanisms (reviewed in 166).

We are relatively early in uncovering the full impact that TEs have had on human disease and genome evolution. Intact mobile elements spread broadly and gave rise to copious degenerate repeats that can be co-opted for the regulation and function of individual genes (17, 35). Only a very small number of TEs remain intact and capable of transposition, but abundant degenerate TEs that can no longer hop remain; for clarity, we refer to the latter as TE-derived sequences (TEDS). TEDS derived from LINE1 (L1) and SINEs (Alu) are the largest class of these interspersed repeats (Figure 1c) and are our main focus here. The challenge is to understand the potential functional raison d’être for millions of these degenerate TEs and to consider how their higher-level genomic organization may relate to their functions.

Below, we briefly review some of the more abundant and/or active TEs and introduce the various known ways that individual TEDS have been co-opted to contribute to the functions of specific protein-coding genes. In Section 4, we discuss emerging evidence for the less established roles of TEDS in broad genomic regulation. For more in-depth information on TEs, we refer readers to several excellent recent reviews (14, 80, 150, 166, 170).

### 3.1. A Brief Introduction to the Major Types of Transposable Elements

**Figure 3:**
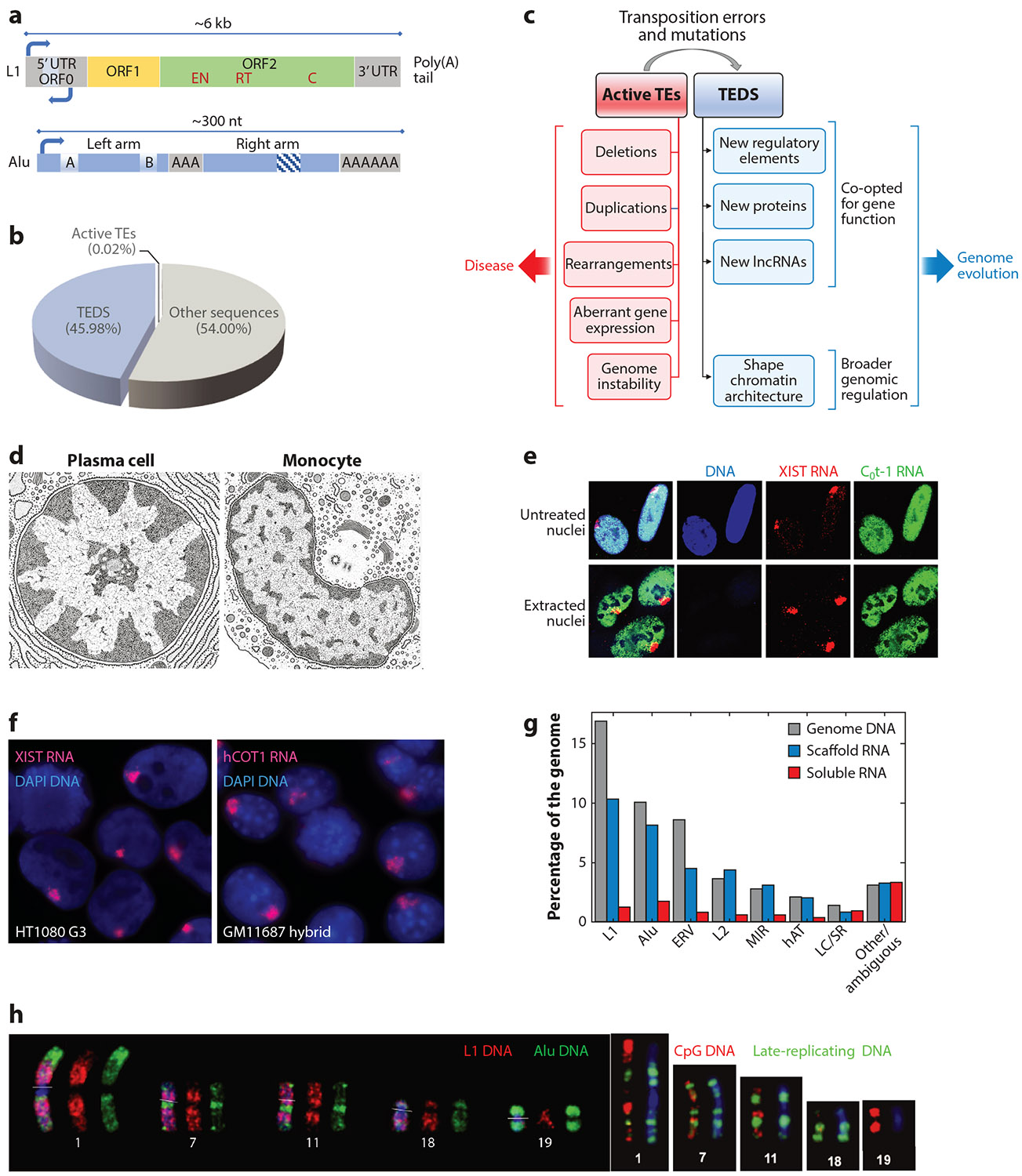
(*a*) Structure of L1 and Alu TEs. (*b*) Pie chart showing the proportions of TEDS, active TEs, and other sequences in the genome. Degenerate TEDS are no longer mobile but vastly outnumber active TEs, which are a tiny fraction of the genome. (*c*) Diagram showing the typically negative effects of TE transposition compared with the positive contributions of TEDS in the function of individual genes, as well as their emerging broader role in nuclear genome architecture. (*d*) Line drawings of plasma cell and monocyte nuclei illustrating how the organization of the condensed heterochromatic compartment and the more open euchromatin differs between cell types. Panel adapted with permission from Reference 26. (*e*) Images of untreated and extracted nuclei showing that both XIST RNA (*red*) and C_0_t-1 RNA (*green*) remain localized and bound with the nuclear scaffold after nuclear extraction and removal of histones and DNA. Panel adapted with permission from Reference 39. (*f*) XIST RNA in HT1080 G3 cells (*left*) and C_0_t-1 RNA in GM11687 hybrid cells (*right*). Similar to how XIST RNA localizes to the inactive X chromosome territory (*left*), human C_0_t-1 RNA strictly localizes on the active human chromosome territory in hybrid cells (*right*). Panel adapted with permission from Reference 73. (*g*) Graph of soluble or scaffold-associated repeat RNAs relative to their abundance in the genome. Repeats sequenced in nuclear RNA are overwhelmingly associated with the insoluble nuclear scaffold. Panel adapted with permission from Reference 39. (*h*, *left*) Examples of L1 and Alu distribution on several human mitotic chromosomes as detected by DNA FISH. (*Right*) The same chromosomes labeled for CpG density and late replication. Left subpanel adapted with permission from Reference 74; right subpanel adapted with permission from Reference 15. Abbreviations: C, cysteine-rich domain; DAPI, 4′,6-diamidino-2-phenylindole; EN, endonuclease; ERV, endogenous retrovirus; FISH, fluorescence in situ hybridization; L1/2, long interspersed nuclear element 1/2; LC, low complexity; IncRNA, long noncoding RNA; MIR, mammalian-wide interspersed repeat; ORF, open reading frame; RT, reverse transcriptase; SR, simple repeats; TE, transposable element; TEDS, TE-derived sequences; UTR, untranslated region.

LINEs are the largest family of retrotransposons and the largest family of repeats in the genome, and L1 is the predominant LINE in humans. The intact L1 is ~6 kb in length and has a bidirectional RNAPII promoter (Figure 3a). Sense transcription produces ORF1p (open reading frame 1 protein), an RNA-binding protein that interacts with L1 RNA, and ORF2p, a protein with endonuclease and reverse transcriptase activities; both proteins are required for retrotransposition (reviewed in 11). Additionally, a second promoter drives transcription in the antisense direction, transcribing the primate-specific ORF0, which produces a small peptide whose function is unknown but which may enhance L1 mobility. ORF0 contains splice donor sites that allow it to form fusion proteins with exons from neighboring genes (46). There are ~560,000 L1 sequences (the bulk of which are TEDS), comprising ~17% of the human genome (83) (Figure 1c). However, only ~7,000 of these L1s have an intact promoter (94), and only ~80–100 evolutionarily recent L1s are intact and capable of retrotransposition (22).

SINEs (reviewed in 170) are the most abundant TEs (and TEDS) in the human genome, and Alu is the largest subfamily of SINEs, accounting for ~10% of the genome (Figure 1c). Unlike L1, Alu is a much smaller TE (~280 bp) and is composed of left and right arms (derived from 7SL RNAs) separated by a poly(A) stretch, with the left arm containing weak RNAPIII promoters (45) (Figure 3a). Alu and other SINEs do not encode reverse transcriptase and thus rely on L1 for retrotransposition (49). There are ~1.15 million Alu sequences in the human genome (83), but as with L1, the vast majority are TEDS, and only a fraction are still capable of retrotransposition (45, 99). Mammalian-wide interspersed repeat (MIR) elements are ancient mammalian SINEs that number ~600,000 (~3%) in the human genome (170). Full-length MIRs are 260 bp long and contain a tRNA-derived left arm, which contains RNAPIII promoters, a central SINE sequence, and a LINE-derived right arm (170). MIR density has been correlated with tissue-specific gene expression (87), and MIRs have been found to function as gene regulatory elements such as insulators (164) and enhancers (86). While our focus here is on Alu and L1, below we briefly introduce a few other, less abundant TEs and provide relevant reviews.

The hominid-specific SINE–variable number tandem repeat (VNTR)–Alus (SVAs) are the youngest family of retrotransposons, with a complex composite structure including a hexamer repeat (CCCTCT), an Alu-like sequence, a GC-rich VNTR, a SINE, and a poly(A) tail (76). While there are relatively few (~6,600) SVA elements in the genome (83), they can be active, but, like Alu, they are nonautonomous, relying on L1 machinery for mobilization (136). SVA insertions have been associated with numerous diseases (132), ~60% are within 10 kb upstream of genes, and evidence suggests they may be involved in gene regulation (135). Interestingly, SVAs are often concentrated in clusters of KRAB–zinc finger protein genes (especially on chromosome 19), which are themselves thought to be involved in the control of TEs (67).

Long terminal repeat retrotransposons are defined by the presence of flanking long terminal repeat sequences, typically flanking two ORFs, gag and pol. They comprise ~8% of the genome and are primarily human endogenous retroviruses (HERVs), remnants of ancient retroviral infections (reviewed in 150). Mutations have rendered most HERVs replication incompetent and largely silent (88); however, the youngest family of HERV-K elements retains the capacity to produce viral proteins and virus particles (82). HERV-K appears to be expressed in most normal tissues (23) but is highly expressed in a variety of cancers, where it may promote tumor growth and metastasis (48). The HERV-H family is also noteworthy because it is highly expressed in human embryonic stem cells, and evidence indicates that it is essential for pluripotency (reviewed in 140).

Numerous families of DNA transposons collectively comprise ~3.6% of the genome (83). They typically have a transposase ORF flanked by terminal inverted repeats and can vary greatly in size (166). Nonautonomous miniature inverted-repeat TEs do not encode a transposase and rely on the machinery of other DNA transposons to mobilize (57). While no longer active (128), DNA transposons have played a significant role in genome evolution in many organisms, including humans, by altering the structure or regulation of specific genes and triggering chromosomal rearrangements (58).

### 3.2. Transposition Activity of Intact LINEs and SINEs and Its Consequences

Less than 0.05% of the millions of TE sequences remain intact and capable of transposition (Figure 3b), and all of them are retrotransposons, including evolutionarily young families of intact LINEs and SINEs. The rate of transposition in humans is low: Approximately 1 in every 17 births carries a new TE integration (59). Since TE activation can have harmful effects, mobile TEs are largely silenced via epigenetic mechanisms, including DNA methylation, histone modifications, and silencing mediated by Piwi-interacting RNA and small interfering RNA (1, 34). While tightly regulated in most somatic tissues, L1 expression and retrotransposition do occur at specific times in development (154). Transposition of L1 is more frequent in early embryogenesis and occurs in specialized cells such as spermatozoa and oocytes (65, 105). Low-level L1 and/or Alu activity may also contribute to somatic mosaicism (92), particularly in the brain (reviewed in 16).

### 3.3. The Downside of Retrotransposition

Integration of a mobile TE in or near a gene will often disrupt normal gene structure or regulation and can also lead to ectopic recombination, chromosomal rearrangements, duplications, or deletions (35, 139) (Figure 3c). TE mobilization has been implicated in a number of neurodegenerative and neuropsychiatric disorders (34, 130), and TEs are frequently dysregulated in cancer (24). L1 expression and retrotransposition can profoundly affect genome stability via DNA damage and replication stress (5, 118) and have been reported to increase in aging and cell senescence, driving interferon expression and inflammation (42).

The bidirectional L1 promoter (Figure 3a) can produce transcripts containing 5′ L1 antisense sequences along with sequences of nearby genes. These chimeric transcripts are produced in several cell types, potentially affecting up to 4% of human genes (40), and some are associated with cancer (27, 95). L1 sequences also contain multiple putative splice sites that can cause aberrant splicing, resulting in disease (e.g., 12, 168).

Like L1, Alu transposition can cause insertion mutations and recombination as well as affect local gene expression and function through a variety of similar mechanisms (10). Alu activity has also been implicated in a number of neurological and other diseases (103, 131).

### 3.4. Some Transposable Element–Derived Sequences Have Been Co-opted for a Role in Local Gene Function

While TE hopping can be deleterious to the host organism, over evolutionary time, mobile TEs gave rise to millions of TEDS, which for poorly understood reasons have persisted to vastly outnumber the active TEs (Figure 3b). Some TEDS have been domesticated for normal gene functions, such as generating new functional regulatory elements, ncRNAs, and proteins (reviewed in 56, 63) (Figure 3c). For instance, more than 20% of regulatory elements in the human genome are TE derived, and more than 85% of these are primate specific (4, 51). Approximately 75% of human genes have at least one Alu sequence, and examples of Alu regulating the function of nearby genes are especially numerous. These include acting as *cis*-acting DNA regulatory elements (e.g., promoters, enhancers, insulators, or TF binding sites) or within mRNAs (in introns or untranslated regions) to influence splicing, nuclear retention, and mRNA stability (reviewed in 170). A recent study showed that some enhancers may use RNA pairing to interact with specific promoters and that almost 40% of these RNA interaction sites overlap Alu sequences (108). In addition, transcription of L1 sequences has enhancer functions that are essential to zygotic genome activation in mouse embryos (107).

TE sequences are also a source for the evolution of new genes (Figure 3c). More than 80% of human long ncRNAs (IncRNAs) contain at least one TEDS, with TEDS comprising ~40% of IncRNA sequences (93). For example, the structural RNAs NEAT1 and XIST, which are responsible for the formation of paraspeckles and X inactivation/Barr body formation, respectively, contain numerous repetitive sequences, some of which may derive from TEs, and which serve as binding sites for proteins essential to their function (54, 169). In addition to IncRNAs, microRNAs and Piwi-interacting RNAs can also be derived from TEs. TEs have also been exapted to create more than 100 new proteins. For instance, CENP-B, which is involved in centromere formation, was derived from a DNA transposon. A variety of proteins important in lymphocyte, placenta, and brain development are TE derived (reviewed in 56).

The studies cited above and numerous others have demonstrated that an interspersed repeat sequence can contribute to the regulation or function of a nearby gene, or as part of the gene itself. However, this does not necessarily attribute functionality to the sea of innumerable repeats interlaced through the whole genome. A major challenge remains to understand whether the abundance of interspersed repeats serves some general genomic function or is mostly evolutionary detritus. This question is the focus of the next section.

## 4. HIGHER-LEVEL ORGANIZATION OF INTERSPERSED REPEATS LINKED TO NUCLEAR COMPARTMENTALIZATION

The collective ~1.6 million Alu and L1 sequences far exceed the protein-coding portion of the genome (~100-fold), so might such abundant repetitive “junk” play a broader role in genome regulation, beyond the level of individual genes? Interspersed repetitive sequences are particularly well-suited to propagate a pattern of chromatin folding across a larger region, using mechanisms such as phase separation of repeat-binding proteins or a unique capacity to interact and form unusual structures (G-quadruplexes, triplex DNA/RNA, etc.). Here, we consider emerging evidence implicating repeats, including repeat-rich RNAs, in higher-level regulation of functional nuclear architecture. In particular, we relate how differences in gene and repeat family distributions are organized across the human karyotype and how this relates to genome regulation within compartmentalized nuclear structure.

### 4.1. The Nuclear Genome Segregates into Large Heterochromatin and Euchromatin Compartments

The nuclear genome is packaged into two large, cytologically distinct compartments (Figures 1f and 3d): condensed, inactive heterochromatin, mostly near the nuclear or nucleolar peripheries, and open euchromatin, which occupies much of the interior nuclear regions in most cell types. It may often be thought that the activity of individual genes explains the visible decondensation evident throughout euchromatin, but it does not. For perspective, it is important to recognize issues of scale. Some (but not all) genes within the decondensed euchromatic compartment will be expressed, but this is a small fraction of the total open chromatin in this region, and packaging changes for individual active genes occur at a much smaller scale than the formation of the compartment. Increasing evidence supports that regional formation of heterochromatin is not driven by the off state of individual genes. For example, during initiation of X inactivation (induced by XIST RNA), the large, condensed Barr body forms before chromosome-wide gene silencing (157), and Polycomb complexes (PRC1) can mediate long-range DNA interactions to form heterochromatin compartments independent of local histone modifications and gene repression (18). Similarly, the initiation of the nuclear heterochromatic compartment forms in two- to four-cell embryos before any cell type–specific changes in gene expression (25, 85).

These cytologically visible nuclear compartments are at a larger scale than structures detected by Hi-C (chromosome conformation capture), which uses DNA cross-linking to investigate sequence organization in nuclei. This approach identifies topologically associating domains (TADs) or sub-TADs and the larger A and B compartments (109). TADs are small intrachromosomal self-interacting regions that are tethered by CTCF binding sites. Notably, more than 95% of mammalian CTCF sites are derived from TEs (SINEs, LINEs, and long terminal repeats), and almost all disease-associated STRs localize with CTCF boundaries (79). Although recent studies have found increasing complexity to Hi-C structures, they are not clearly linked to euchromatin/heterochromatin packaging. However, TADs are bundled into A and B compartments (of variable size, ~1 Mb or more), which correspond to euchromatin (A) and heterochromatin (B1/B2) bundles. While each A and B compartment reflects packaging well above the gene level, the cytological-scale nuclear compartments are built by congregation of numerous A-with-A and B-with-B Hi-C compartments.

We hypothesize below that the repetitive sequences that make up much of the fabric of a chromosomal region are related to, and likely play a role in, forming heterochromatin versus euchromatin regions. Before discussing how this relates to the karyotypic organization of gene and repeat sequences, we summarize the important point that repeats are abundant not only in DNA but also in nuclear RNA.

### 4.2. Abundant Repeat-Rich Scaffold RNA Physically Supports Open Chromatin in Nuclear Territories

Recent evidence indicates that the unexplained length of pre-mRNA, IncRNA, and long intergenic ncRNA (lincRNA), much of which is repeat sequences, serves a structural role in nuclear chromosome territories. Specifically, this RNA supports open euchromatin structure. C_0_t-1 DNA (the most highly repetitive genomic fraction; Figure 1a) is typically used as a cold competitor to mask the “uninteresting” repeats. However, one study used labeled C_0_t-1 DNA as a probe to examine repeats in RNA by molecular cytology and made several unanticipated findings (73). Since repeat-rich introns are generally rapidly degraded upon cotranscriptional splicing, the bright, robust signal indicated a surprising abundance of repeats in RNA throughout the nucleus (Figure 3e). C_0_t-1 RNA is excluded from the peripheral heterochromatin and the inactive X chromosome coated by XIST RNA. Analysis of a mouse/human hybrid cell with one human chromosome showed that the human C_0_t-1 RNA remains tightly localized to the human chromosome territory, unlike for excised introns, appearing remarkably similar to the XIST RNA territory that covers the inactive X chromosome territory (Figure 3f). Surprisingly, the localized C_0_t-1 RNA territory remained after prolonged transcriptional inhibition but could be rapidly dispersed by disrupting a nuclear scaffold protein, causing chromatin condensation (e.g., 98), which suggested that it could be a euchromatic structural RNA. This idea was supported by several studies showing that disruption of nuclear RNA causes cytological chromatin condensation and implicating HnRNP-U (heterogeneous nuclear ribonucleoprotein U)/SAF-A (scaffold attachment factor A) or similar proteins, which have both DNA- and RNA-binding domains, as being involved (reviewed in 115).

To identify the RNA sequences involved in nuclear chromosome structure, a biochemical fractionation procedure was developed to isolate nuclear scaffold RNAs that remain insoluble after removal of histones and DNA (39) (Figure 3e). The procedure extracts most nuclear RNA and leaves just 15% that cofractionates with known architectural RNAs, XIST RNA, and NEAT1 RNA [which forms the scaffold for nuclear paraspeckles (32)]. The insoluble RNAs that remained with the nuclear scaffold are composed almost entirely of long, repeat-rich C_0_t-1 RNAs (pre-mRNAs, IncRNAs, and lincRNAs), and repeat RNA sequences are found almost entirely in the nuclear scaffold fraction (Figure 3g). This C_0_t-1 heterogeneous nuclear RNA is associated with known nuclear scaffold/matrix RNA-binding proteins [matrin 3, NuMa (nuclear mitotic apparatus), and SAF-A] and forms an RNA-binding protein meshwork that promotes open chromatin. A recent report also directly showed that matrin 3 binds repeat RNAs, particularly L1 antisense RNA, and that the disruption of RNA binding (by a mutation that causes amyotrophic lateral sclerosis) causes aberrant chromatin condensation (171). Thus, evidence supports that long, repeat-rich “junk” RNA is integral to maintaining euchromatin structure and that the repeats within this RNA may play a key role.

This evidence that long, repeat-rich heterogeneous nuclear RNAs function in maintaining open chromatin suggests an unanticipated role for intron sequences in euchromatin structure around active genes. Introns often allow for alternative splicing; however, this does not explain their excessive length [some over 50 kb (142)] or why 80–90% of pre-mRNA sequence (and many IncRNAs) is noncoding and replete with repeats. Repeat-rich intronic RNAs, IncRNAs, and lincRNAs might help stabilize the epigenetic state of euchromatin and may also explain recent findings that revealed exceptionally long-lived RNAs, including pre-mRNAs and IncRNAs, in nuclei of terminally differentiated mouse neurons (173); these RNAs appear to be highly analogous to human C_0_t-1 scaffold RNAs (for commentary, see 104).

Interspersed repeat RNAs may also play protective roles throughout the nuclear genome in response to stress, adding to the evidence of a function for repeats in the stress response, as established for HSat3 RNA (detailed in Section 2.4.1). For example, in response to stress, Alu elements are expressed from their own RNAPIII promoter, and the transcripts directly bind RNAPII, repressing global transcription (116). The subsequent widespread loss of transcription would otherwise cause deleterious chromatin condensation, but, surprisingly, new transcription of long, intergenic, repeat-rich C_0_t-1 RNAs is induced upon stress, including in response to osmotic shock (159) or reversible transcriptional arrest (39). These extremely long intergenic transcripts have been termed DOGS (downstream of genes) and suggested to play a role in protecting chromatin from collapse by maintaining euchromatic C_0_t-1 RNAs. This idea was supported by the observation that despite the arrest of genic transcription during the stress response, the new intergenic transcription maintained C_0_t-1 scaffold RNA levels (39), and this was required to avoid chromatin collapse. These findings highlight that different components of the noncoding repeatome play a role in response to stress.

### 4.3. Cytogenetic Bands Reveal High-Level Organization of Genes and Interspersed Repeats

High-level organization of the genome sequence is apparent in the cytogenetic banding pattern of mitotic chromosomes (Figure 1e), which reflects large blocks of chromatin (typically 5–10 Mb) with distinct properties. Giemsa–trypsin staining produces a pattern of alternating dark G-bands and light R-bands, which show certain differences in sequence content and biochemical properties (15) (Table 1). Some evidence suggests that staining differences reflect different folding of DNA loops into a proposed AT queue [along the chromosome axis (138)] and/or greater compaction of G-band versus R-band chromosomal DNA (9, 68).

Important for our focus here is that the density and types of interspersed repeats, as well as genes, also show a corresponding segmental distribution: SINE (Alu) elements are enriched in R-bands, and LINE (L1) elements are enriched in G-bands (74, 100) (Figure 3h). There are corresponding differences in CpG island distribution and late versus early replication between chromosome bands (15) (Figure 3h; Table 1). Here, we discuss the segmental organization of genes and repeats primarily in relation to G- and R-bands; however, we note that this binary categorization is a simplification. As we briefly discuss below, some studies identify five categories of bands based on the depth of Giemsa staining (64). Similarly, T-bands are a subtype of light bands with an especially high density of genes and CpG islands (15). All of this reflects a segmental organization of the genome sequence.

The functional significance for this cytological-scale organization has not been widely considered. Banding patterns are invariant between people (and cell types), but this does not necessarily mean that this organization is unrelated to genome regulation. Relevant to this is that segmental patterns of gene and repeat organization within synteny blocks are largely conserved (e.g., 38), even though primary sequences of SINEs and LINEs are not. In addition, mobile SINEs do not preferentially integrate into regions where they are most commonly found (90), and active L1s appear to prefer integration into R-bands (153) instead of G-bands, where L1 TEDS are enriched. Thus, enrichment of LINEs in late-replicating segments and SINEs in gene-rich early-replicating regions may be evolutionarily favored and conserved across species.

**Figure 4:**
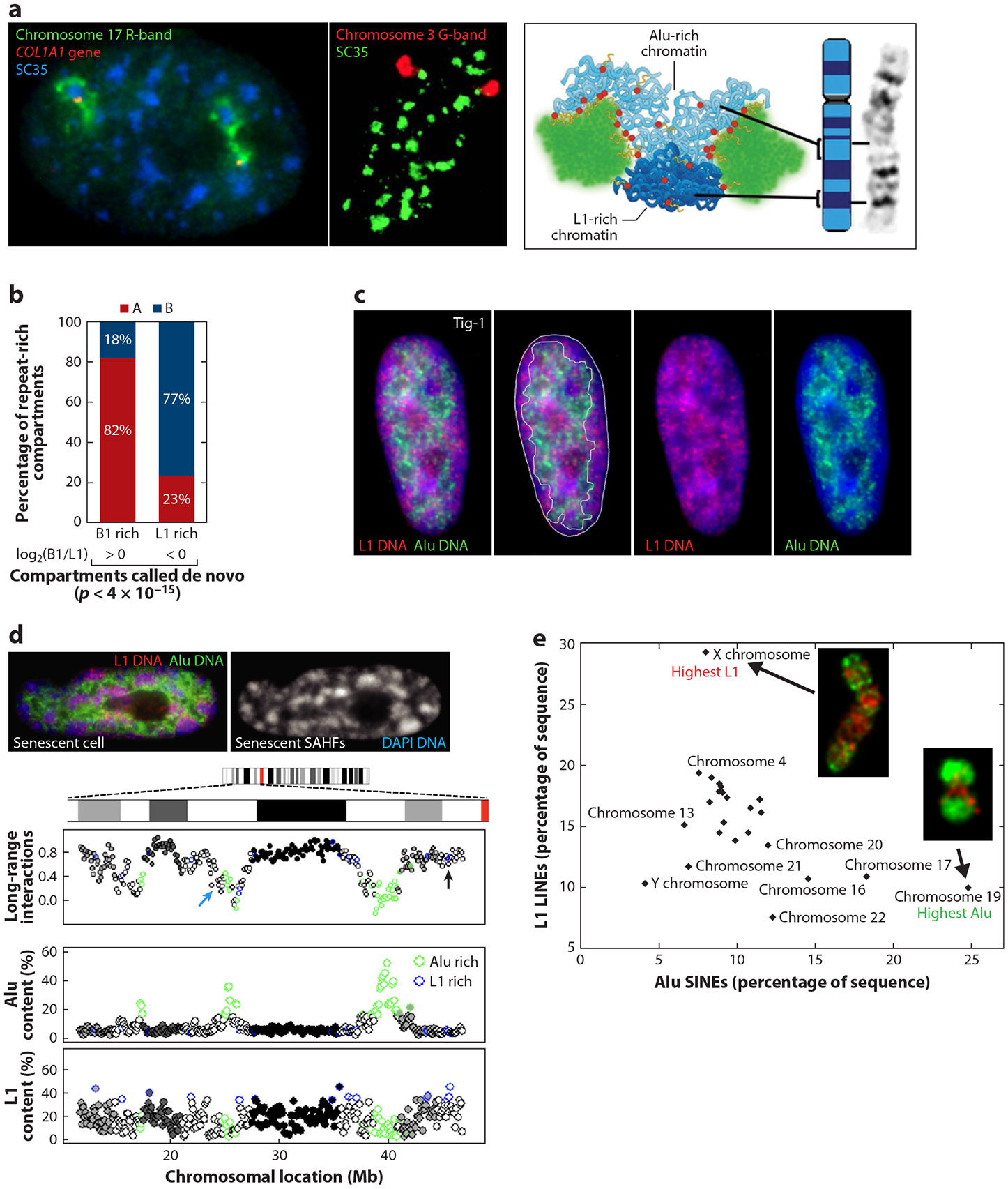
(*a*, *left*) DNA FISH image showing R-band DNA (17q21; *green*) with a *COL1A1* gene (*red*) that closely associates with nuclear speckles (stained for splicing factor SC35; *blue*). (*Center*) DNA FISH image showing that G-band DNA (*red*) is more condensed at the nuclear periphery (SC35 speckles; *green*). (*Right*) Model showing that gene- and Alu-rich R-band DNA (*light blue*) is more intimately associated with nuclear speckles than L1-rich, gene-poor G-band DNA (*dark blue*). Panel adapted with permission from Reference 143. (*b*) Percentages of repeat-rich compartments as examined by Hi-C approaches. The most SINE-rich (B1 in mouse) compartments are primarily A compartments (active), whereas the most LINE-rich (L1) are B compartments (inactive). Panel adapted from Reference 112 (CC BY 4.0). (*c*) DNA FISH images for L1 (*red*) and Alu (*green*) in human fibroblast nucleus, with the green signal outlined in the second image to show Alu depletion at the periphery (DAPI; *blue*). Panel adapted with permission from Reference 74. (*d*, *top*) DNA FISH images of a senescent fibroblast nucleus for L1 (*red*) and Alu (*green*) DNA. On the right, DAPI DNA shows dense heterochromatin foci (SAHFs). (*Bottom*) Ideogram of 50 Mb of chromosome 4 aligned with the graph, showing changes in long-range (*>*10 Mb) intrachromosomal Hi-C interactions between senescence and growing cells (log_2_). Each dot represents 100 kb and is colored according to Giemsa-band designations; the black arrow indicates a condensing L1-rich R-band, and the blue arrow indicates an R-band containing Alu-rich peaks that resist condensation. Also shown are the percentages of Alu and L1 content across the same region, with the highest (90th percentile) Alu content outlined in green and the highest L1 content outlined in blue. Panel adapted with permission from Reference 74. (*e*) Relative contributions (as percentages of total chromosome sequence) for L1 and Alu in all human chromosomes. The insets show the X chromosome and chromosome 19 stained for L1 (*red*) and Alu (*green*) DNA. Panel adapted with permission from Reference 74. Abbreviations: DAPI, 4′,6-diamidino-2-phenylindole; FISH, fluorescence in situ hybridization; L1, long interspersed nuclear element 1; LINE, long interspersed nuclear element; SAHF, senescence-associated heterochromatic focus; SINE, short interspersed nuclear element.

The essential co-opted functions of Alu elements with individual genes (e.g., TF binding, covered in Section 3) may partially explain why Alus are enriched in gene-rich bands. However, this still raises the question of why genes—predominantly housekeeping genes—would be nonrandomly clustered in large chromosome regions, corresponding to light R-bands. A strong rationale for this clustering comes from the finding that genes and pre-mRNA metabolism are nonrandomly organized within interphase nuclei. Within the euchromatin compartment, many active genes preferentially distribute in ~10–20 nuclear speckles (also known as SC35 domains) rich in pre-mRNA metabolic and splicing factors (reviewed in 30, 75), and gene and Alu-rich R-bands are in spatial proximity to nuclear speckles, whereas adjacent dark bands are frequently condensed at the periphery (143) (Figure 4a). The entire R-band in Figure 4a appears to be decondensed (66), including regions not containing active genes, while other regions enriched for active genes are expressed in close proximity to nuclear speckles (143). Gene clustering around nuclear hubs that promote efficient gene expression further provides a functional rationale for the large-scale clustered regional distribution of coding genes on chromosomes. This demonstrates a fundamental relationship between the segmental organization of the linear genome on chromosomes and the structural organization of the functional genome in nuclei. It also explains why Alu-rich DNA is more densely clustered around these same structures (29, 74) and why Alu-rich DNA is highly correlated with regions of highest expression in the interphase nucleus (29).

Since Alu SINEs can contribute to the functions of individual genes, their enrichment in gene-rich R-bands may simply reflect evolutionary conservation. However, their presence in gene-rich regions would not require that Alu be strongly depleted from other regions (discussed in Section 4.4), and the 1.1 million Alu TEDS that are more concentrated in the gene-rich segments suggest greater Alu density than is easily explained by individual gene regulation. Hence, there remains a question as to whether regional densities of Alu may be evolutionarily conserved for additional, perhaps broader, contributions.

### 4.4. Highly Alu-Rich Segments Resist Condensation, and Alu Is Depleted from L1 Heterochromatin

A priori, there is no clear reason why the density of Alu and L1 TEDS has an inverse distribution in alternating multi-megabase chromosome segments, but this may well reflect distinct contributions to nuclear compartmentalization in interphase. LINE repeats have long been suggested to play a role in forming heterochromatin, including the spread of silencing on the inactive X chromosome in female cells (31, 114). L1 is enriched in lamina-associated domains (~1–10-Mb DNA segments abutting the nuclear lamina and peripheral heterochromatin compartment), and increased L1 density is also seen in B compartments at the nuclear periphery (reviewed in 106, 147) (Figure 4b,c). While heterochromatin is prevalent in the nuclear periphery of most cell types, the overall nuclear pattern of heterochromatin is distinct and characteristic of different cell types (Figure 3d). It has long been our view that these cytological patterns of chromatin architecture reflect the framework for coordinated genome-wide regulation of specific cell types. The heterochromatin compartment will contain both constitutive heterochromatin, which likely nucleates the compartment, and facultative heterochromatin, containing genes silenced in specific cell types.

Since LINEs are prevalent throughout the genome, their modest enrichment in gene-poor G-bands and heterochromatin could simply be because transposable LINEs were selected against in gene-rich regions (R-bands). However, recent work has provided direct evidence for a functional role of degenerate L1s in forming heterochromatin. L1 RNA, which is transiently expressed from L1 TEDS in very early embryogenesis, is required for the de novo formation of the constitutive heterochromatin compartment (before cell type–specific gene regulation begins) (85, 112, 113, 129). L1 RNA interacts with L1 DNA to help nucleate a compartment marked by specific chromatin modifications, and then the RNA must be silenced to maintain the heterochromatic state (106, 149). Hence, L1 RNA induces changes that establish a stable heterochromatic state, similar to XIST RNA triggering X chromosome heterochromatin. We note that this role of L1 RNA as an inducer of heterochromatin formation is a distinct mechanism from C_0_t-1 and L1 antisense RNA physically supporting open euchromatin (Section 4.2).

Recent evidence indicates that the depletion of Alu-rich peaks, not just L1 density, may be an important factor influencing whether there is condensation of a region (74). In many primary human fibroblasts, the peripheral heterochromatin appears to be more clearly delineated by Alu depletion than byL1 enrichment (74) (Figure 4c). The condensed L1-rich Barr body, at the core of the inactive X chromosome, also excludes Alu-rich DNA. In addition, when senescent cells completely reorganize peripheral heterochromatin into senescence-associated heterochromatic foci (SAHFs), Alu-rich regions are excluded from these condensed bodies as well (74) (Figure 4d). Hi-C data analysis revealed long contiguous Alu peaks as the most striking variance in repeat distribution, and the Alu-peak region consistently countered chromosome compaction (as indicated by increased long-range intrachromosomal interactions). Figure 4d shows a quantification of L1 and Alu (in 100-kb bins) that demonstrates two additional points. First, L1 density on this chromosome (chromosome 4) is similarly high across both R- and G-bands, which can show similar structural changes (e.g., the black arrow indicates a condensing L1-rich R-band); in contrast, the R-band containing Alu-rich peaks (blue arrow) resists this change (condensation). Second, results also show that architectural interactions change in unison across whole large (~5–15 Mb) chromosome segments; for example, DNA throughout the whole darkest G-band shows increased long-range interactions, suggesting that the band’s architecture changes as a single structural unit.

Evidence indicates that constitutive heterochromatin forms in the darkest G-bands (G-positive bands 75–100), which are the most L1 rich but also the lowest in Alu. Data from an earlier study of five different band classes (64) indicated that SINEs (as a percentage of total sequence) essentially double between the darkest and lightest bands (from 8.4% to 15.6%), whereas LINEs decrease by ~25% (from 25.1% to 19.2%). Marked differences in Alu and L1 enrichment are also seen for certain whole chromosomes that similarly differ in their propensity to form heterochromatin. The X chromosome has the highest L1 content (Figure 4e), although the L1 density is lower in the pseudoautosomal region that escapes gene silencing (8). In marked contrast, chromosome 19 is strikingly Alu rich and low in L1, has the highest gene density, and consistently resides in the euchromatic nuclear interior (78). Chromosome 19 is also unusual in that it does not form a heterochromatic SAHF in senescent cells (74). Furthermore, chromosome 19 is an outlier in that it encodes a concentration of more than 250 zinc finger regulatory proteins (44), many of which are regulated by SVA elements. Based on the singular nature of the repeat content and gene content of chromosome 19, we suggest that this whole small chromosome may be uniquely maintained as constitutive euchromatin in different human cell types (and potentially in higher primates with conserved synteny).

### 4.5. Future Directions: The Language of Chromosome Bands and Small Common Words in Developmental Regulation

In our view, we have only seen the tip of the iceberg when it comes to the meaningful biology in the sea of repeat-rich “junk” that makes up so much of the human and many higher genomes. Clearly specific repeats have often been co-opted in ways that influence the structure or function of individual genes. Granted, repeat content does not simply correlate with organismal complexity, and the abundant TE-derived repeats could be mostly evolutionary detritus. However, if we assume this, and repeats en masse are generally screened from studies, then we could overlook a potentially fundamental aspect of genome and developmental biology. Evidence cited above provides precedent that certain repeat families are expressed or otherwise play a role in specific contexts, such as in response to stress, during developmental changes, or specific diseases. Hence, if we do not look in different cellular contexts for changing expression, structural interactions, or epigenetic modifications across the repeatome, we will not find them.

There are many next questions, and we will end by highlighting two with relevance to the language of chromosome bands. As noted above, there are different flavors of bands, with different depths of staining, which likely reflect sequence content. Learning to decode the language of segmental chromosome organization may well require distinct approaches that seek to identify patterns of organization across larger regions than typically studied. This may also prove important in genome regulation during development. As illustrated in Figure 3d, the cell type–specific patterns of cytological genome organization likely reflect changes in the heterochromatic versus euchromatic state of certain facultative regions. Changes in the expression of specific genes can certainly occur within the euchromatin compartment, regulated at the histone or nucleosome level. However, since many cell type–specific genes are in L1-rich dark bands, these are likely facultative heterochromatin, and thus regions that switch compartmentalization in different cell types (reviewed in 106), which may relate to the sequence differences reflected in distinct subtypes of chromosome bands. We suggest that many cell type–specific genes will be regulated within regions that can change chromatin state, and not only by mechanisms of individual gene expression. This concept has fundamental importance for genome regulation that merits more investigation.

There has been more research into Alu and L1 TEDS, so these have been our focus here. However, this is a simplistic view, because there is more complexity of repeats in the noncoding genome. Perhaps most overlooked and poorly studied are abundant small “common words”—interspersed simple sequence repeats (i-SSRs). In addition to TF binding sites, SSRs can form unusual DNA structures that could influence chromatin folding (81). Hence, we make a distinction between locus-specific small tandem repeats (e.g., triplet repeats, discussed in Section 2.1) because common i-SSRs are interwoven throughout the genomic fabric and thus could influence its regional packaging. Comprising ~3% of the human genome (101), some i-SSRs are remarkably prevalent, for unknown reasons. Most notably, the 9-mer word ATATATATA occurs 100 times more frequently than the median 9-mer word (119), which could be related to an AT queue in the chromosome axis (138). Chromosomal distributions of i-SSRs have not been well-characterized; however, there is evidence that SSR enrichment in a region may correlate with its regulation. For example, a count of all 9-mer words in the genome revealed a striking 11-fold enrichment of GATAGATAG that was interspersed across the 10-Mb region of the X chromosome that escapes X inactivation (119). While the twofold-lower density of L1 TEDS in this region is often cited as evidence for L1 function in silencing, the SSR content is largely overlooked. In this review, we have not focused on this less studied feature of the genome, but i-SSRs could prove important to understanding the language of the genome in chromosome biology.

## Figures and Tables

**Table 1:** Characteristics of chromosome bands

	G (Giemsa dark)	R (reverse, or Giemsa light)
Sequence	AT rich	GC rich
CpG content	CpG poor	CpG rich
Gene density	Gene rich	Gene poor
Gene type	Housekeeping	Tissue specific
Replication timing	Earlier	Later
TE density	LINE enriched	SINE enriched

Abbreviations: LINE, long interspersed nuclear element; SINE, short interspersed nuclear element; TE, transposable element.

## CATEGORIES OF HUMAN TANDEM REPEATS

- Satellites: Satellites are generally very large arrays (megabases) of repeated units that can range from 5 bp to several hundred base pairs and collectively comprise 6.2% of the human genome. There are two main types: centromeric satellites, which make up the main functional centromere of each chromosome, and pericentromeric satellites, which are adjacent to some but not all human centromeres (reviewed in 2, 121).
- Macrosatellites: Microsatellites have a repeat unit size of >100 bp (typically ~1–6 kb), in arrays ranging from a few kilobases to hundreds of kilobases in length; each is usually found in only one or two loci in the genome. Although their sequences are unrelated, they tend to be CpG rich, often produce noncoding and/or coding RNAs, and tend to be regulated by DNA methylation (reviewed in 52).
- Minisatellites: Also called variable number tandem repeats (VNTRs), minisatellites are typically defined as having repeat units of 6–100 bp (158) and highly variable array lengths (typically from 0.5 kb to several kilobases). There are approximately 1,000 minisatellites in the genome, located mostly near the ends of chromosomes.
- Microsatellites: Also called short tandem repeats (STRs), microsatellites have repeat units of 1–6 bp repeated ~6–30 times; ~500,000 of them are scattered across the genome, constituting ~2.5% of the human genome (66, 83). Microsatellite arrays are highly polymorphic in length, primarily due to a replication error called strand slippage, as well as misalignment at meiosis and unequal crossover. Changes in the copy number of STRs at specific gene loci have been linked to many diseases, as discussed in Section 2.1. Their abundance and variability have also made them important tools in molecular diagnostics, population studies, and forensics (reviewed in 66). In Section 4.5 we make a distinction between localized STRs and interspersed simple sequence repeats (i-SSRs), which are extremely high-copy-number repeat “words” dispersed throughout the whole genome.
